# A framework for scaling up health interventions: lessons from large-scale improvement initiatives in Africa

**DOI:** 10.1186/s13012-016-0374-x

**Published:** 2016-01-29

**Authors:** Pierre M. Barker, Amy Reid, Marie W. Schall

**Affiliations:** 1 Institute for Healthcare Improvement, Cambridge, USA; 2 University of North Carolina at Chapel Hill, Chapel Hill, USA

**Keywords:** Scale-up, Spread, Adaptive design, Sustainability, Large-scale spread, Quality improvement

## Abstract

**Background:**

Scaling up complex health interventions to large populations is not a straightforward task. Without intentional, guided efforts to scale up, it can take many years for a new evidence-based intervention to be broadly implemented. For the past decade, researchers and implementers have developed models of scale-up that move beyond earlier paradigms that assumed ideas and practices would successfully spread through a combination of publication, policy, training, and example.

Drawing from the previously reported frameworks for scaling up health interventions and our experience in the USA and abroad, we describe a framework for taking health interventions to full scale, and we use two large-scale improvement initiatives in Africa to illustrate the framework in action. We first identified other scale-up approaches for comparison and analysis of common constructs by searching for systematic reviews of scale-up in health care, reviewing those bibliographies, speaking with experts, and reviewing common research databases (PubMed, Google Scholar) for papers in English from peer-reviewed and “gray” sources that discussed models, frameworks, or theories for scale-up from 2000 to 2014. We then analyzed the results of this external review in the context of the models and frameworks developed over the past 20 years by Associates in Process Improvement (API) and the Institute for Healthcare improvement (IHI). Finally, we reflected on two national-scale improvement initiatives that IHI had undertaken in Ghana and South Africa that were testing grounds for early iterations of the framework presented in this paper.

**Results:**

The framework describes three core components: a sequence of activities that are required to get a program of work to full scale, the mechanisms that are required to facilitate the adoption of interventions, and the underlying factors and support systems required for successful scale-up. The four steps in the sequence include (1) *Set-up*, which prepares the ground for introduction and testing of the intervention that will be taken to full scale; (2) *Develop the Scalable Unit*, which is an early testing phase; (3) *Test of Scale-up*, which then tests the intervention in a variety of settings that are likely to represent different contexts that will be encountered at full scale; and (4) *Go to Full Scale*, which unfolds rapidly to enable a larger number of sites or divisions to adopt and/or replicate the intervention.

**Conclusions:**

Our framework echoes, amplifies, and systematizes the three dominant themes that occur to varying extents in a number of existing scale-up frameworks. We call out the crucial importance of defining a scalable unit of organization. If a scalable unit can be defined, and successful results achieved by implementing an intervention in this unit without major addition of resources, it is more likely that the intervention can be fully and rapidly scaled. When tying this framework to quality improvement (QI) methods, we describe a range of methodological options that can be applied to each of the four steps in the framework’s sequence.

## Background

Major variations exist in the dimensions of quality of care—safety, efficiency, effectiveness, timeliness, patient centeredness, and equity [1]—which can be seen as a failure to equitably scale up excellent care, that is, getting what we know works to everyone who needs it [2]. For some, excellent care is within reach; for many others, in predictable patterns, it is not within reach because of the systems we have built. Scaling up ideas, tools, programs, and policies is not straightforward [3]. To address this challenge, we explore the scale-up process and the many previous efforts to identify the common components of scale-up in order to build a framework of practical use.

While the terms “spread” and “scale-up” have been used interchangeably in some implementation science literature, some argue that “spread” refers to the adoption and replication (with little modification) of an intervention within a health system, whereas “scale-up” addresses the system/infrastructure issues that arise during full-scale implementation [4]. Others have distinguished the institutionalization of scale-up (“vertical” scale-up) and the expansion from replication to scale-up (“horizontal” scale-up) [5]. We have not assigned definitions to these terms; rather, we describe three main components of scaling up: using a clear sequence of activities needed to take interventions to scale, articulating the context and environmental factors that will foster scale-up of best practices, and describing the infrastructure that is required to support scale-up.

Although several existing frameworks for scale-up address these three core components to different extents, none provides actionable guidance on how to integrate all three components to take an intervention from concept to full scale. A key element of this model is the concept of the “scalable unit”—defining a microsystem or a mesosystem that can be replicated as the intervention is scaled up. Microsystems have been described extensively as an organizing model to improve the functionality of health care [4] but have not been explicitly integrated into a purposeful plan for scale-up. The framework also describes how a range of quality improvement and other implementation strategies can be used to progress towards full scale at each step in the sequence. To accelerate the pace of getting to full scale, we also recommend adaptive design features at each step that can accommodate and learn from different contexts inevitably encountered during the scale-up process.

## Methods

To develop our framework, we first reviewed the extensive literature on scale-up and spread and identified six approaches that specifically addressed three core components for achieving results at scale: a step-wise journey from an idea to full-scale implementation, environmental factors that foster adoption, and infrastructure required to support scale-up. We then re-examined the work of the quality improvement community—primarily Associates in Process Improvement and the Institute for Healthcare Improvement—that included a series of evolving ideas on sequencing, adoption mechanisms, and infrastructural support of scale-up. Finally, we reflected on two national-scale improvement projects that the Institute for Healthcare improvement (IHI) undertook in Africa that were testing grounds for early iterations the framework and allowed us to refine it further.

### Review of existing sequential scale-up approaches

To better understand our framework’s location within existing thinking about sequential scale-up, we identified other scale-up approaches for comparison and analysis. We searched for systematic reviews of scale-up in health care, reviewed those bibliographies, spoke with experts, and reviewed common research databases (PubMed, Google Scholar) for papers in English from peer-reviewed and “gray” sources that discussed models, frameworks, or theories for scale-up from 2000 to 2014. We used the following search terms: “scale,” “spread,” “scale-up,” “health systems,” “health care,” “health services,” “models,” and “framework.” Our review was designed specifically to seek out published frameworks that used a sequential approach to scaling up health interventions. From our inquiries and screening of a large number of abstracts (>16,000), we identified 45 models or frameworks for in-depth review. Through this process, we identified six sequential scale-up approaches, and we compare and contrast these frameworks with the scale-up framework presented in this paper.

### Review of quality improvement-based approaches

Guided by their principal scientific partner, Associates in Process Improvement (API), and building on the essentials of process improvement developed in industry, the IHI has developed over the past 20 years a series of models and frameworks for diffusion and scale-up of improvement approaches that have been taught and tested in multiple settings. We reviewed the contributions of Plan Do Study Act (PDSA), Breakthrough Series Collaborative, and Framework for Spread to scale-up. Both the sequential scale-up approaches referenced above and the previous work by API and IHI contribute strongly to the framework presented here.

## Results and discussion

### Six existing frameworks for sequential scale-up from the literature

The literature on achieving results at scale describes various approaches, taking into account factors at the smallest scale, including details of the intervention itself; factors at the largest scale, including the larger socio-political and economic context; and myriad factors in between, including variables related to the implementing health systems, communities, and practitioners [5–11]. This approach accommodates multi-level interventions that address the complexities of the environment and interacting systems. In addition, several articles point to lessons from outside of health care, including from social movements and complex adaptive systems [12–14]. We review six existing frameworks that advocate a sequential approach—a particular ordering of phases for successful scale-up and spread—and provide practical guidance for how to work with organizations, health systems, and communities to implement and scale up best practices.

The three core categories we sought to understand better—the journey from an idea to full-scale implementation, environmental factors that foster adoption, and infrastructure required to support scale-up—are reflected to varying degrees in the six frameworks for achieving results at scale that we studied (Table 1). All six frameworks include phased activities used to move new interventions or pockets of best practices to full scale [15–23]. To move through the phases, methods used include theory testing in different settings at a small scale and deep inquiry to understand the context and planning before moving forward. All frameworks promoted the use of data to reflect and improve the future design of the work and acknowledge factors that impact spread (e.g., intervention characteristics and the will for change). Some highlight the importance of looking ahead to build needed infrastructure to support full-scale implementation and advocate testing resource requirements during smaller tests of implementation. Others rely more on pre-planning, predictions of resource needs, and feedback after implementation.

**Table 1:** Review of frameworks for scaling up health interventions

Frameworks	Sequential scale-up plan	Adoption influences and infrastructure
*Implementing Best Practices* C*onsortium (15*,*16)*	Preliminary setup phase, a test-of-concept phase, further testing in different environments, and an implementation scale-up phase to get to full scale; theory-based approach that tests the applicability of the intervention in different contexts before scaling	Outlines eight principles that support change including perception of benefits, change agent, resource support for the change agent, leadership support, staff motivation, small-scale testing using success to motivate, clear implementation ownership, and getting going by not delaying first steps
*Expandnet (17–19)*	Alignment to the local practices and contexts in the setup phase, and testing and learning from different contexts as the intervention starts to scale up, feeding the information learned into the final scale-up plan; theory-based approach that tests the applicability of the intervention in different contexts before scaling	Emphasis on understanding attributes of the innovation, the organization, the resource team and the larger social, political, economic, and institutional environment
*WHO/Massoud (20)*	Preliminary setup phase, a test-of-concept phase in a representative “slice” of the system, and exponential increase of these slices to fill out the areas of full scale through further testing in different environments; theory-based approach that tests the applicability of the intervention in different contexts before scaling; a major contribution from Massoud is the notion of planning from the outset with scale in mind and initial testing in a network of facilities across multiple layers of the system	Use of evidence of success as a mechanism for advocacy and will building, and creating a receptive environment for taking an intervention to full scale; suggest using leaders from successful early test phases of the work to become the advocates and local champions to drive the scale-up phases of the work
*Management Systems International (21)*	Planning, establishing pre-conditions for scaling up, and implementation; accounts for, and anticipates the needs of, different contexts through deep inquiry into local conditions	Highlights the need for pre-work, stage setting, and engagement that will support successful scaling up, especially in terms of attaining necessary resources and buy-in through advocacy methods
*Consolidated Framework for Implementation Research (22)*	Planning, engaging, executing, and reflecting/evaluating; accounts for, and anticipates the needs of, different contexts through deep inquiry into local conditions	Five areas to consider: intervention characteristics, inner setting, outer setting, individual characteristics, and the implementation process
*Yamey (23)*	Phased delivery strategy as one of six success factors that needs to account for and anticipate needs of different contexts through deep inquiry in to local conditions as well as using a phased approach	Outlines six areas that influence successful scale-up, including attributes of the tool/service being scaled up, of the implementers, of the community, of the socio-political environment, of the research environment, and the delivery strategy

### Non-sequential scale-up approaches

A number of *non-sequential* approaches to taking an intervention to full scale have also been described [24]. These include policy changes and executive mandates (e.g., the banning of traditional birth attendants to increase women’s attendance in facilities), campaigns that saturate coverage of specific geographies over a short period of time (e.g., vaccination drives, deworming), more complex “campaign” approaches that disseminate evidence-based bundles and a “playbook” of implementation strategies (e.g., IHI’s 100,000 Lives Campaign), and a rapid mobilization approach used when spread is required in emergency situations (e.g., vaccination against a new virus like H1N1 or cholera). These approaches all have an important role in implementing health interventions and should be considered in the final phase of the sequence of activities described in our framework.

### Evolution of models and frameworks that use quality improvement as the “engine” for change

**Fig. 1:**
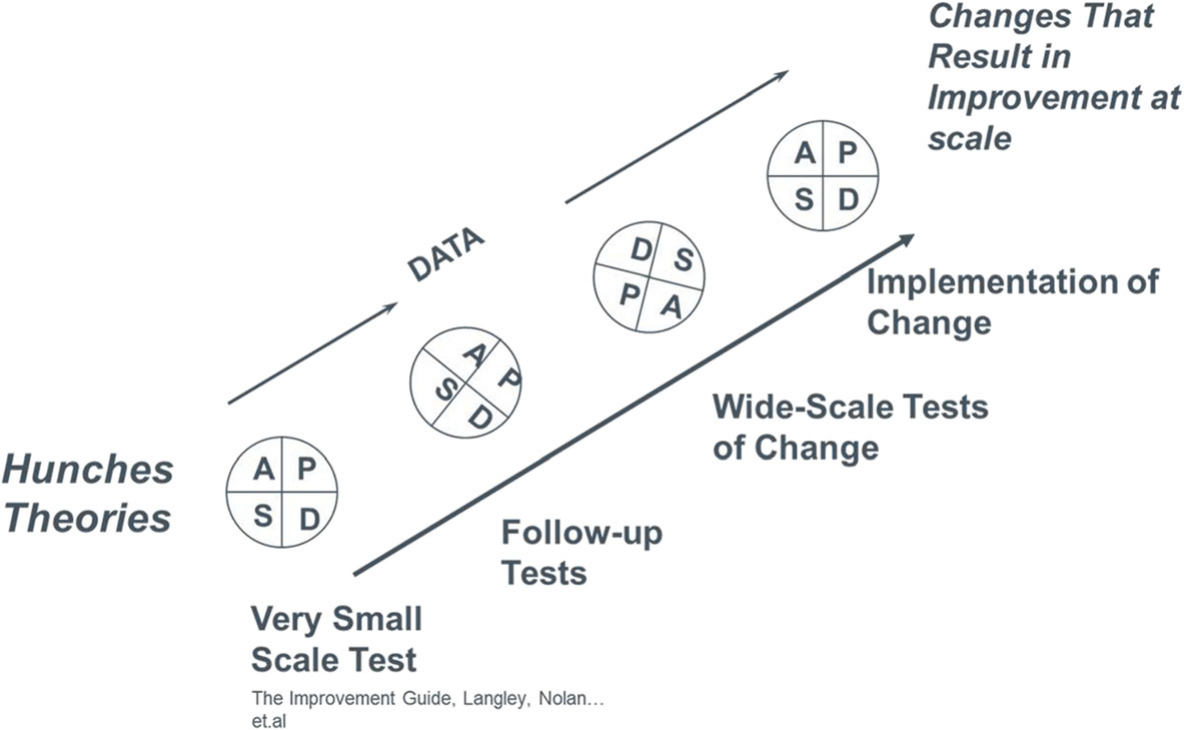
Rapid-cycle improvement. Integral to the Model for Improvement, an improvement approach developed by Associates in Process Improvement, rapid-cycle improvement is a disciplined way to iteratively test changes in a process at a larger and larger scale (Langley GJ, Nolan KM, Nolan TW, Norman CL, Provost LP. *The improvement guide: a practical approach to enhancing organizational performance.* San Francisco: Jossey-Bass Publishers; 2009). Based on a theory about what change will lead to improvement, a change is first tested at a very small scale, e.g., with one clinician and one patient, using the Plan-Do-Study-Act method. Based on the results of each cycle, further tests are planned or the change may be abandoned

At the heart of the quality improvement (QI) method is rapid-cycle testing using the Shewhart Plan-Do-Study-Act (PDSA) cycle [25], which ensures that ideas for change are tested and adapted for local context. The notion of using PDSA to “ramp up” the implementation process into broader and more diverse environments, as proposed by Associates for Process Improvement (Fig. 1), was a breakthrough in understanding how to apply QI to the scale-up process. This concept provides the essential requirement for an adaptive design that can accommodate different contexts that are encountered as the intervention is scaled up.

The QI approach is central to the IHI “Breakthrough Series,” or BTS, model [26]—a structured learning system that brings a number of teams from different settings together to accelerate the implementation of improved processes within and across participating organizations. Quality Improvement methods were also a core element of IHI’s 100,000 Lives Campaign, which used a rapid national dissemination (campaign) approach to reach thousands of hospitals across the USA [27].

**Fig. 2:**
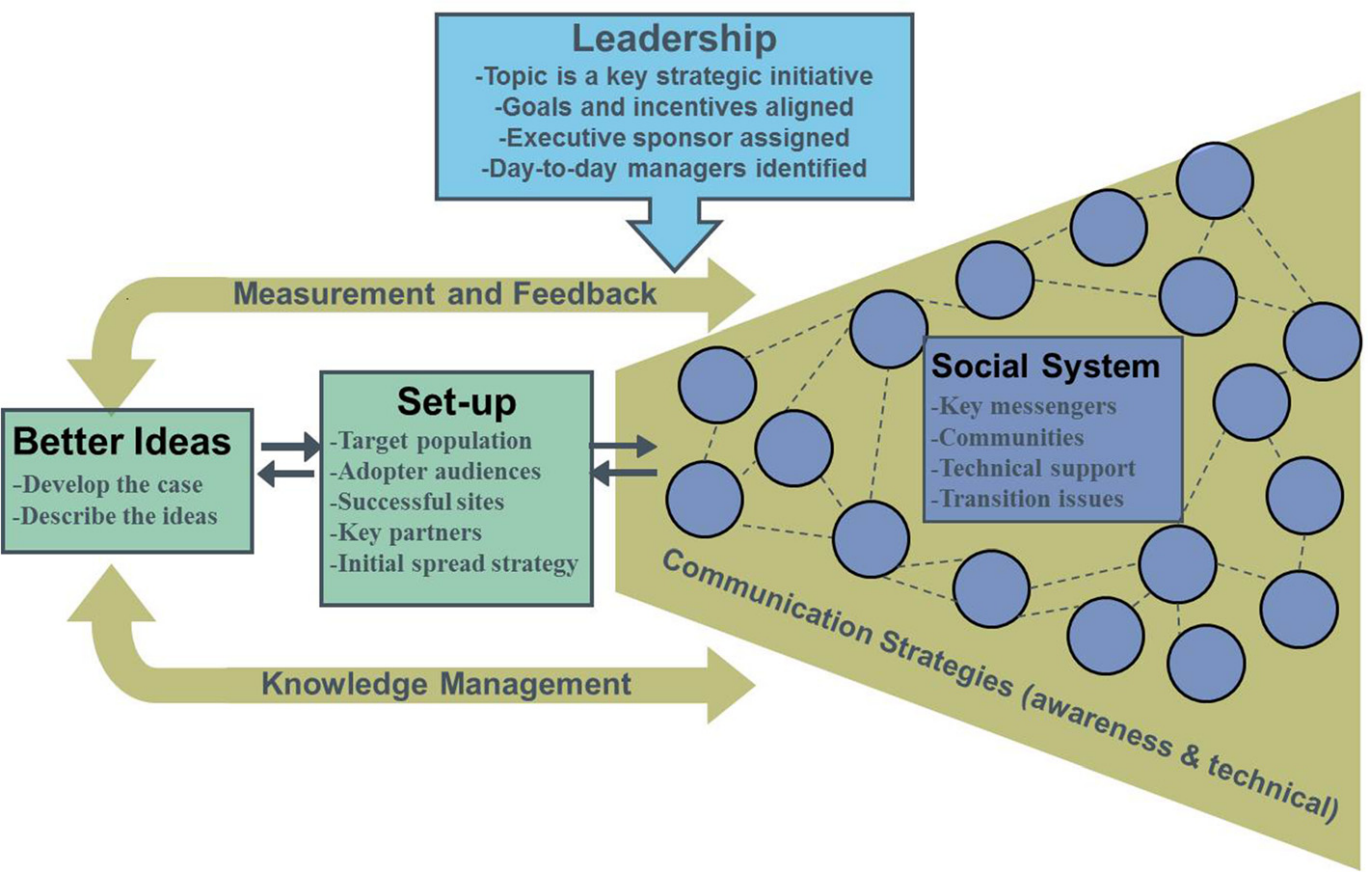
IHI Framework for Spread. IHI’s earlier Framework for Spread [28] identifies six areas that have been shown to contribute to successful spread: the role of organizational or governmental leadership in setting the agenda for change, aligning incentives, and establishing accountability; the development of better ideas and practices that demonstrate the relative advantage of such practices over the old way; the development and use of communications channels and messages; the strengthening the social system; the use of data to guide spread; and the refinement of the spread effort as needed, based on feedback from the field and data on the performance of the system

Efforts to understand the determinants of spread resulted in IHI’s early Framework for Spread [28], which was designed to help organizations, primarily hospitals and health systems, expand the impact of their work from pilots or small-scale interventions to larger areas within their organizations or communities (Fig. 2). The Framework for Spread brought attention to the determinants of spreading good practice (i.e., social science, organizational structure, and network properties) [29–33]. The framework also emphasized the role of leadership behavior as a key determinant of success in spreading evidence-based interventions.

Our evolving understanding of the social and environmental determinants of going to full scale—addressed in the new framework—reflects two realities of carrying out this kind of work: first, the need to account for the factors that are required both to promote adoption of the changes and to support scale-up; and second, the need to design a phased plan from the outset with full-scale implementation in mind. Many pilots cannot progress to scale-up because the specifications of the pilot cannot be replicated at scale.

### A new framework

**Fig. 3:**
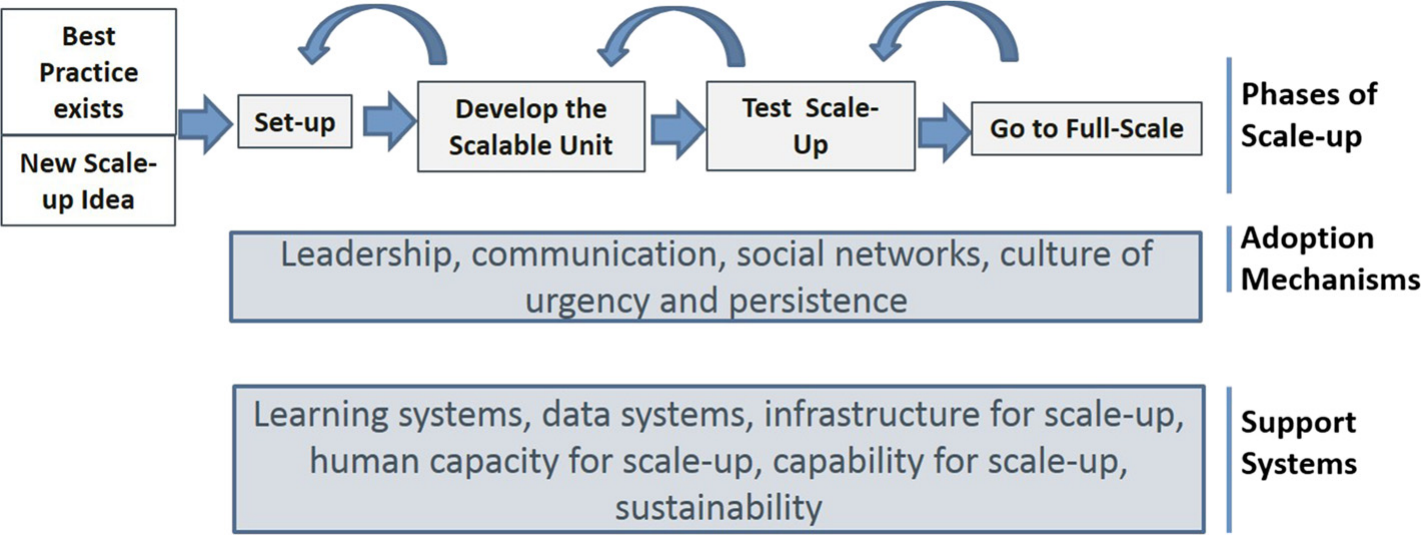
IHI Framework for Going to Full Scale. The IHI Framework for Going to Full Scale addresses the phases of going to full scale and the adoption mechanisms and support systems needed to achieve large-scale programming. The elements of the framework include the phases of going to full scale (i.e., *Set-up*, *Develop the Scalable Unit*, *Test of Scale-up*, and *Go to Full Scale*); adoption mechanisms (i.e., leadership engagement, communication methods, leveraging social networks, and building a culture of urgency and persistence); and support systems needed to achieve large-scale programming (i.e., a learning system that connects adopters and experts, a data system to support measurement for improvement, infrastructure such as IT, equipment, etc.), building capability through training and support, and building reliable process that support sustainability

The Framework for Going to Full Scale (Fig. 3) defines four phases required to get to full scale: (1) *Set-up*, which prepares the ground for introduction and testing of the intervention that will be taken to full scale; (2) *Develop the Scalable Unit*, which is an early test and demonstration phase, (3) *Test of Scale-up*, which spreads the intervention to a variety of settings that are likely to represent contexts that will be encountered at full scale; and (4) *Go to Full Scale*, which unfolds rapidly to enable a larger number of sites to adopt and/or replicate the intervention. We discuss the importance of designing in sustainability at all phases. While this sequence reflects a logical progression from conception to full scale, in reality, the phases may not be linear; rather, they may be more organic and iterative, with streams of work initiated at different times and progressing at different rates.

#### Setup

This phase establishes an entry point for the planned intervention into the existing health system. This phase includes a clear articulation of what needs to be scaled up and defines the ambition for “full scale.” During this phase, initial test sites, early adopters, and potential “champions” of the intervention are identified.

#### Develop the scalable unit

This phase develops the “scalable unit”—the smallest representative facsimile of the system targeted for full-scale implementation. The purpose of this phase is to intensively test local ideas for best-practice implementation so that the interaction among all parts of this representative sub-system can be understood. An important outcome is the generation of a set of context-sensitive strategies and interventions (change package) that can be further tested and refined in a broader range of settings. This change package will drive rapid improvement of performance during the *Go to Full Scale* phase.

The scalable unit is typically a small administrative unit (e.g., sub-district/district or clinical ward/division) that includes key infrastructural components and relationship architecture that are likely to be encountered in the system at full scale. If the ambition of scale is large (e.g., county, province, health system), a scalable unit could comprise multiple levels of care and the communities that are served by a large health system, or a divisional unit of care in a hospital setting or large clinic system.

Initial testing can be done at a single site if that site represents the scalable unit; however, if the scalable unit requires inclusion of multiple sub-units (e.g., clinics and a hospital in a district), an adaptation of the IHI Breakthrough Series (BTS) Collaborative model [26] can be used to accelerate learning and build the initial change package. When scaling to a nation or a region, the scalable unit itself may be very large and complex (e.g., large health district, large Accountable Care Organization). In that case, a sub-system (e.g., sub-district, hospital, referral clinics/communities) can be tested first, before broadening the work to include all parts of the scalable unit. The IHI “Idealized Design” process proposed that this early testing phase could itself comprise several iterative steps required to deliver a change package that would be ready for further dissemination through the BTS mechanism [34].

#### Test of scale-up (i.e., testing the set of interventions to be taken to scale)

The underlying theory of change and the change package from a successful early demonstration need to be tested in a broader range of settings before going to full scale. Also, during this phase, we test necessary infrastructure (e.g., data systems and supply chain) required to support full-scale implementation and build the human capacity and capability (e.g., leadership, managerial, and frontline capacity needed to support the method being used to scale up). This phase is an important opportunity to build the belief and will of leaders and frontline staff to support the changes. As with the *Develop the Scalable Unit* phase, the BTS model can be an effective mechanism to accelerate the adoption of new ideas and generate a more mature change package that can be used for full-scale implementation across a range of contexts.

#### Go to full scale

This is a rapid deployment phase in which a well-tested set of interventions, supported by a reliable data feedback system, is adopted by frontline staff on a larger scale. The focus is on rapid uptake of the intervention through replication. While some adaptation of the intervention to local environments will always be required, there is less emphasis on new learning in this phase. Significant will, knowledge, experience, and well-tested infrastructural support and capacity need to be in place before moving to this phase; the determinants of adoption as reflected in the IHI Framework for Spread (i.e., intrinsic properties of the change, the social environment, and the network properties) are well established.

Experience with this approach suggests that the rate of expansion can be exponential (i.e., not linear) by a multiple of 5 of the number of units involved in the scale-up (e.g., 1–5–25–125–625, etc.). The actual multiple can vary depending on the complexity of the intervention and the complexity of the environment. In South Africa, the scale-up included 3 districts, then 10, and then all 52 for the *Develop the Scalable Unit*, *Test of Scale-up*, and *Go to Full Scale* phases, respectively. In Ghana, the number of units scaled went from 35 sub-districts to 265 to 289 to 554. (See the case reports below.)

#### Adoption mechanisms

The environment for change and psychology of change are crucial factors that foster or hinder the pace and extent of adoption of the intervention. Rapid scale-up will not occur in an unreceptive environment. At each step of the scale-up process, the design of the intervention needs to be closely attuned to the social beliefs and health system practices, taking account of and closely integrated with policies, protocols, and other health system structures. We outline five areas that impact adoption.

Identifying factors that affect adoption should start in the *Set-up* phase with understanding the health system’s infrastructure, culture, size, and strength of its underlying social system [28]. Understanding the psychology of change and whom to target in the different phases is crucial to success of scale-up; in the *Set-up* phase, the different segments of the target adopter population (e.g., leaders, caregivers, populations) and early adopters are identified.

##### Better ideas

Everett Rogers identifies several key characteristics of the intervention itself [33] that are key determinants of the scalability of the intervention and its rate of adoption by the broader community. These include the evident superiority of the intervention, its simplicity, and its alignment with the culture of the new implementers.

##### Leadership

The crucial role of leadership at all levels for system transformation is well described [35], and the capacity for leading large-scale change needs to be developed as part of the scale-up process. Leaders can be coached to understand the difference between simply raising awareness of a new practice and what it takes to lead and ensure its broad adoption. To get results, IHI has promoted a number of systematic approaches to engage leaders in their key role of guiding and supporting large-scale change [36, 37].

##### Communication

The early demonstration phase (*Develop the Scalable Unit*) is a crucial time for communicating the value of the intervention to both leadership and the implementers (frontline staff). Providing real-time data is a powerful way to draw attention and garner support for the next phase of scale-up. Early adopters and charismatic frontline staff who have successfully implemented the intervention in this phase become powerful advocates for the intervention to their peers. During this phase, the “early majority” of Rogers’ Diffusion of Innovations curve [33] are targeted with these communications, while in the *Test of Scale-up* phase, the audience includes the “late majority,” preparing the ground for more rapid and extensive scale-up.

##### Policy

The identification and/or development of regulatory or administrative policies are an important environmental factor that can either inhibit or expedite the adoption of specific interventions. Policies that create negative financial or procedural incentives function as barriers to adoption by making the desired actions more difficult to test and sustain. Conversely, policies associated with positive incentives can enhance the motivation to change behavior.

##### Culture of urgency and persistence

Leaders of initiatives that are intended to achieve results at scale should consider several key questions when they begin their initial planning, including why others would want to join the effort and whether there is a glaring gap in performance or an urgent need [38]. Responses to these questions serve as a barometer for the amount of will and energy needed to stay the course in bringing interventions to—and achieving results at—full scale. While the levels of will and energy may fluctuate over the course of an initiative, the other adoption mechanisms described above can help to enhance adopters’ ability to sustain their efforts.

#### Support systems

This phased scale-up approach needs a supporting infrastructure. The following components of support should be considered in a scale-up design from the outset:

##### Human capability for scale-up

The expanding QI capability needs of a scale-up initiative should be anticipated early in the *Set-up* phase. While frontline staff can be trained in basic QI methods, scale-up will require team leaders who can use change management approaches to guide and mentor teams at the front line and improvement specialists who can lead and design QI-based programs for those who need additional training. The project needs be able to communicate quantitative results and the underlying stories of success and challenge. Data managers need training in analytic and reporting capabilities that are best suited to QI methods (e.g., run charts and statistical process control).

##### Infrastructure for scale-up

Ideally, scale-up can be achieved primarily through redesign rather than addition of new resources, such as hiring new staff, but the *Develop the Scalable Unit* phase may reveal resource constraints that cannot be overcome through system redesign. Common structural considerations include additional tools (e.g., checklists, data capture systems), communication systems (e.g., materials and messages, mentoring relationships, structured programs), and key personnel (e.g., data capturers, quality improvement mentors) that are specifically assigned to enable better system performance.

##### Data collection and reporting systems

Having reliable systems that track and provide feedback on the performance of key processes and outcomes is essential to any scale-up initiative. While some ad hoc data collection is essential to track new ideas, particularly in the demonstration and scale-up testing phases, large-scale implementation cannot occur or be sustained unless routine data systems are accurate, complete, and timely. In addition, data that tracks key processes and outcomes that are targeted by the intervention need to be shared frequently with frontline staff and system leaders to inform ongoing improvement.

##### Learning systems

Large-scale change requires a mechanism for collecting, vetting, and rapidly sharing change ideas or interventions. The *Develop the Scalable Unit* phase is the most intensive period of innovation, usually resulting in a large array of change ideas that are being tested and require vetting. During this phase, change ideas that are shown to result in improved performance are assembled into a change package. The BTS model is an effective way to share knowledge between units that are undertaking similar work [39]. Proven change ideas, tested in a variety of settings, that are assembled into the change package during the early phases can be disseminated with confidence and little further modification when the initiative goes to full scale.

##### Design for sustainability

Sustainability is a key design consideration throughout the three activity phases of getting to full scale—*Develop the Scalable Unit*, *Test of Scale-up*, and *Go to Full Scale*. The learnings about sustainability at each phase should be built into the expanding change package. The activities associated with sustainability are well described [40] (e.g., high reliability of the new processes, inspection systems to ensure desired results are being achieved, support for structural elements, ongoing learning systems), and their purpose should be to ensure that the system cannot revert to its prior state of performance. In addition, leadership commitment is required to continue to nourish and replenish these key support elements for scale-up. To sustain the scale-up process, leaders need to commit to a learning system that includes the continuous feedback of data to identify and close gaps in performance [41].

#### Methods of implementation

While a range of methods [38] can be deployed to achieve the aims of each of the phases of the Framework for Going to Full Scale (Table 2), the Model for Improvement [42] is a foundational element of adaptive design that we believe is required to address improvement in the different contexts encountered throughout scale-up. The *Set-up* phase gathers information about the change and the system within which the change will be taken to full scale; the *Develop the Scalable Unit* phase uses structured improvement methods to learn deeply from a small number of sites; the *Test of Scale-up* phase uses methods that engage a larger number of sites in testing the intervention under a wider set of contexts and settings; and the *Go to Full Scale* phase uses methods that have been shown to be effective in large-scale initiatives.

**Table 2:** Methods of implementation that can be used with each scale-up phase

Phase	Setup	Develop the scalable unit	Test of scale-up	Go to full scale
Methods	• Model for Improvement • Surveys • Brainstorms • Expert meetings • Scans • Site visits • Interviews	• Model for Improvement • Idealized design • Collaborative learning (e.g., adaptation of Breakthrough Series [BTS] Collaboratives)	• Model for Improvement • Deployment and refinement of change package • Site redesign • Collaborative learning • Change agents	• Model for Improvement • Extension agents • Affinity groups • BTS Collaboratives • Wave sequence • Campaigns • Hybrid approaches

#### Case examples

##### Context

The centralized and integrated health systems typical of African countries build off a policy framework that is founded on evidence-based knowledge. Those policies are implemented through guidelines, protocols, and associated clinical training and supported by resources required to deliver those programs. Often, programs are also supported by reporting systems to track performance. Recent specific efforts to scale up comprehensive interventions directed at saving lives of infants and children in Africa have not consistently shown evidence of improved outcomes [43, 44]. These failures underscore the major challenges faced when taking existing evidence-based interventions and promising new interventions to full scale rapidly within a variety of different contexts. We describe two case examples—one from South Africa and one from Ghana—that used a sequential approach, accompanied by spread and infrastructural support efforts, to take a set of interventions to full national scale within a few years.

##### South Africa: improving perinatal PMTCT of HIV

South Africa has more HIV-infected people than any other nation and more than 30 % of pregnant women infected with HIV. The prevention of mother-to-child transmission (PMTCT) program was grafted onto South Africa’s well-attended antenatal care service platform, offering, in a primary care setting, the multi-step PMTCT processes of care: testing pregnant mothers for HIV, initiating antiretroviral treatment, and testing babies for evidence of infection at 6 weeks of age. The South African PMTCT program was launched reluctantly by the existing political administration, which may have affected the evolution of the PMTCT scale-up [3]. Given the lack of familiarity with QI methods in South Africa at the time, and the political ambivalence about addressing the HIV epidemic, the project was designed initially as a demonstration of effectiveness of a scalable intervention, without knowing if it would be scaled.

The set-up phase lasted two and a half years because of a struggle to assemble the political will and leadership required to ensure that the Department of Health (DoH) led the project. The *scalable unit* was the health district, but the initial testing and demonstration work was done in self-contained “wedges” within three health districts; each wedge included a district hospital and its 25–30 feeder primary care clinics. These initial demonstrations provided crucial evidence of effectiveness of the approach (rapid improvement in process performance), experience for district managers, supervisors and provincial leaders of how to lead and manage using QI methods, and a demonstration to central health system planners of the effectiveness of QI approaches for improving HIV. With support from local technical partners, the national DoH then led a *test of scale-up* of this approach by initiating QI learning collaboratives in five more districts across the country. This provided opportunities for further refinement of the package of implementation strategies, testing of data systems, indicators and collection tools, and, importantly, the opportunity for the government to assume ownership of the process through its own testing of the process. Following these successful tests, the South African DoH took full leadership and responsibility for taking the program to *full scale*, using a set of implementation strategies that led to a decline in HIV transmission rates from 19 % in 2005 to <5 % in 2010 [45]. To get to full scale (52 districts across the country), the intervention expanded from 3 districts (*Develop the Scalable Unit*) to 8 (*Test of Scale-up*) to 52 (*Go to Full Scale*), demonstrating the opportunity to scale exponentially when using this approach. The innovation and spread methods used were the Model for Improvement, collaborative networks (*Develop the Scalable Unit*, *Test of Scale-up*), and campaign (*Go to Full Scale*).

##### Ghana: national scale-up of MCH programming

In Ghana, a country of 23 million in West Africa, a similar sequence of scale-up unfolded, except that national scale-up was designed from the outset. In the case of Ghana, efforts were underway to reach the country’s Millennium Development Goals (MDGs) 4 and 5 (i.e., reduce child and maternal mortality, by 60 and 75 %, respectively) [46]. A national health systems improvement initiative was introduced in Ghana in 2008 to supplement and accelerate Ghana’s existing maternal and child health (MCH) programs and efforts to reach its MDGs. A set of evidence-based maternal and child survival interventions already existed. The purpose of the QI project was to improve the reliable application of those clinical interventions. Partnering with a faith-based health system, National Catholic Health Service (NCHS), IHI introduced QI methods to more effectively implement an evidence-based package of clinical interventions developed by the Ghana Health Service (GHS).

In Ghana, the district was the scalable unit. The district was made up of sub-districts that included a hospital and other facilities, including primary care clinics. Using a phased design, the initiative scaled exponentially from the north of the country to the south. The sequential design and exponential scale-up approach have been described elsewhere [47]. Starting with the sub-district teams, each district management team built capability to support the work. In the *Test of Scale-up* phase as the project scaled across three northern provinces, the project required significant redesign as it became apparent that more training was required to build sufficient local capability for using QI methods among district and regional supervisors and high-level leaders to support the fast-moving scale-up design. Successful demonstrations of improvement for key maternal and child outcomes (e.g., hospital-based deaths) were actively disseminated, and these results became a prime motivator for increasing adoption of the approach by the regional and then national leadership. The disadvantage of not having the Health Ministry and the GHS involved deeply in the design from the outset is that it was not possible to work on sustainability mechanisms (e.g., a national plan to replenish QI mentors who are lost through turnover) until the Ministry developed major interest near the end of the project. To get to full scale, the project scaled exponentially from 35 sub-districts in the *Develop the Scalable Unit* phase, to 265 sub-districts in the *Test of Scale-up* phase, to 554 sub-districts in the *Go to Full Scale* phase over 6 years. The Breakthrough Series Collaborative design was the primary method of learning and spread at each phase of the project. The project reached more than 80 % of all public and faith-based hospitals in the country.

## Conclusion

The framework proposed in this paper integrates three dominant themes that exist to varying extents in a number of existing scale-up frameworks: a sequential approach to getting to full scale, the factors that enhance the receptivity of the environment into which the intervention is being scaled, and the system-level factors that are required to support scale-up. The framework adds three substantial and practical contributions to the current understanding on this topic:

- First, while a number of existing frameworks mention, to varying extents, the sequential approach, adoption mechanisms, and infrastructure supports, our framework argues that all three are key elements of design for getting to full scale.
- Second, we call out the crucial importance of defining the scalable unit. If a scalable unit can be defined, and successful results achieved in this unit without major addition of resources, it is likely that the intervention can be rapidly scaled (so long as adoption and infrastructural issues are addressed). Health systems are much less automated and arguably more heterogeneous compared to equivalent large business enterprises; as a result, we cannot jump from the prototype scalable unit to wide-scale replication, as in other industries.
- Third, when tying this framework to QI methods, we describe a range of methodological options that can be applied to each of the four phases of the framework.

All the frameworks that we studied included mechanisms for accommodating context into the design. About half the frameworks used deep situational exploration to familiarize themselves with the environment and worked with local stakeholders to formulate designs that were context sensitive. The other half, in line with IHI’s adaptive design approach, used the learnings from early demonstration models to iteratively inform and modify the design as the project scaled. While all frameworks proposed that learnings from the implementation experience be incorporated into subsequent design, we argue that rapid and successful scale-up would benefit from a formative rather than a summative approach, providing as many opportunities as possible to reflect and redesign throughout the process.

The case studies illustrate that real-life experience of implementing a scale-up initiative will rarely follow a set design. In the South African example, there was no scale-up plan at the start of the project, only an intention to create a scalable unit—a compelling demonstration of how an existing health district could achieve high performance results through simple redesign, not addition of resources. Once the will had been established, the *Test of Scale-up* phase was used to further develop the model, averting possible unanticipated challenges associated with going straight to full-scale implementation. In Ghana, the scale-up design was repeatedly modified as the various phases of the scale-up encountered unexpected problems or results were not as good as expected within the planned timeframe.

While the cases described are from low- and middle-income country settings and the external frameworks we reviewed are likewise formulated from experiences primarily in lower-resource settings, we expect that the underlying principles are equally applicable in all settings. The two cases described involve maternal and child health topics, implemented in low-resource settings; while the cases are based on broad principles of health system change generalizable to other health topics, these assumptions need to be tested.

The scale-up framework, using QI methodology, is not the only way large-scale progress can be achieved in health outcomes. Indeed, much progress has been made in the past two decades, with dramatic decreases in maternal and child mortality attributable to economic advances, policy changes, and advances in vaccines and clinical care. However, the reliable implementation and rapid scale-up of known evidence-based interventions remains a challenge. Our two cases can be compared to other major efforts to implement large-scale maternal and child health programming through more conventional, non-sequential approaches—policy and training, i.e., UNICEF’s Accelerated Child Survival and Development (ACSD) program on maternal and child health care [44], and a cluster randomized trial of the effects of the Integrated Management of Childhood Illness (IMCI) strategy on childhood mortality and nutrition in a rural area in Bangladesh [48].

The case examples in this paper suggest that a sequential approach using the components of the Framework for Going to Full Scale may provide guidance about how to design and implement large-scale improvement efforts. We look forward to wider testing of the new framework in various settings and with different types of interventions, to contribute to the knowledge and practice of achieving results at full scale for health care improvement efforts.

## Abbreviations

ACSD: Accelerated Child Survival and Development
API: Associates in Process Improvement
BTS: Breakthrough Series
DoH: Department of Health
GHS: Ghana Health Service
IHI: Institute for Healthcare Improvement
IMCI: Integrated Management of Childhood Illness
MCH: maternal and child health
MDGs: Millennium Development Goals
NCHS: National Catholic Health Service
PDSA cycle: Plan-Do-Study-Act
PMTCT: prevention of mother-to-child transmission
QI: quality improvement
